# A Country on the Verge of Malaria Elimination – The Kingdom of Saudi Arabia

**DOI:** 10.1371/journal.pone.0105980

**Published:** 2014-09-24

**Authors:** Michael Coleman, Mohammed H. Al-Zahrani, Marlize Coleman, Janet Hemingway, Abdiasiis Omar, Michelle C. Stanton, Eddie K. Thomsen, Adel A. Alsheikh, Raafat F. Alhakeem, Phillip J. McCall, Abdullah A. Al Rabeeah, Ziad A. Memish

**Affiliations:** 1 Vector Biology Department, Liverpool School of Tropical Medicine, Liverpool, United Kingdom; 2 Public Health Directorate, Ministry of Health, Riyadh, Kingdom of Saudi Arabia; 3 College of Medicine, Alfaisal University, Riyadh, Kingdom of Saudi Arabia; Tulane University School of Public Health and Tropical Medicine, United States of America

## Abstract

Significant headway has been made in the global fight against malaria in the past decade and as more countries enter the elimination phase, attention is now focused on identifying effective strategies to shrink the malaria map. Saudi Arabia experienced an outbreak of malaria in 1998, but is now on the brink of malaria elimination, with just 82 autochthonous cases reported in 2012. A review of published and grey literature was performed to identify the control strategies that have contributed to this achievement. The number of autochthonous malaria cases in Saudi Arabia decreased by 99.8% between 1998 and 2012. The initial steep decline in malaria cases coincided with a rapid scaling up of vector control measures. Incidence continued to be reported at low levels (between 0.01 and 0.1 per 1,000 of the population) until the adoption of artesunate plus sulfadoxine-pyrimethamine as first line treatment and the establishment of a regional partnership for a malaria-free Arabian Peninsula, both of which occurred in 2007. Since 2007, incidence has decreased by nearly an order of magnitude. Malaria incidence is now very low, but a high proportion of imported cases, continued potential for autochthonous transmission, and an increased proportion of cases attributable to *Plasmodium vivax* all present challenges to Saudi Arabia as they work toward elimination by 2015.

## Introduction

The recent global increase in malaria control efforts has contributed to major reductions in the burden of the disease [1], [2]. Since 2007, four countries have eliminated malaria and been certified by the World Health Organization (WHO) as malaria free [3]. Today, 34 countries, including the Kingdom of Saudi Arabia, are actively attempting to eliminate malaria[4].

Malaria control in Saudi Arabia was initiated in 1948 by the Arabian American Oil Company (ARAMCO) in the Eastern province, primarily to protect employees living around the oases [5]. This programme was used by the Saudi Arabian government as the template for a national malaria programme in 1952, [5], which targeted malarious districts across the kingdom and was designed to protect pilgrims en route to the holy sites of Mecca and Medina. Saudi Arabia joined the WHO global malaria eradication effort in 1963 and, by the early 1970s, transmission was arrested in the Eastern and Northern provinces, eliminating malaria in the Palaearctic ecozone [6]. Despite this success, Saudi Arabia switched from malaria eradication to control in 1977 [7], following the worldwide abandonment of the goal of global malaria eradication.

Today in Saudi Arabia, malaria persists in the provinces of Aseer and Jazan [8], both bordering the Republic of Yemen. Following a series of outbreaks (of which the worst was in 1998), malaria control was intensified and the goal of malaria elimination in Saudi Arabia was re-established in 2004, with an elimination target of 2015. After nearly a decade of activity, progress towards this goal is reviewed in this report. Successful strategies and continued challenges are highlighted and discussed so that other countries may benefit from lessons learned in Saudi Arabia.

## Methods

### Data Sources

Data for this review were identified by literature searches of PubMed and general searches using the Google and WHO search engines. Search term strings included ‘malaria’ AND ‘Saudi Arabia’. The bibliographies of selected documents were used to identify additional data and information sources. The National Malaria Control Programme provided access to archived annual reports and statistical data. Malaria data was obtained from the Ministry Of Health annual statistical reports (1980–2012) [9]. Population data was obtained from the Central Statistical Office. Due to the limited number of data sources available, no formal exclusion criteria were used.

### Analysis

Population figures were extracted from the national censuses in 1992, 2004, and 2010. Population numbers for the interim years were estimated by calculating the average annual population growth between censuses and applying this accordingly. Malaria incidence was calculated as the total number of autochthonous cases divided by the total population.

## Findings

### Vectors and Epidemiology of Malaria

Records of vectors and epidemiology of malaria in Saudi Arabia are limited. A total of 15 Anopheles species have been recorded, five of which are known to be competent malaria vectors: *An. arabiensis, An. sergentii, An. stephensi*, *An. superpictus* and *An. culicifacies* [8]–[14]. *An. arabiensis* is currently the only known vector of malaria in Saudi Arabia [10], [11], with low reported sporozoite rates (<1%) [11], [12]. Abundance of *An. arabiensis* typically peaks following the rains, when multiple breeding sites appear [10], although there is some evidence from Jazan to suggest that irrigation may contribute to malaria risk [12]. With only 40–42% of blood meals in *An. arabiensis* females being of human origin [11], [12], vector populations are clearly supported primarily by other host species, most likely domestic cattle, a preferred host of *An. arabiensis* throughout its range. As *An. arabiensis* rests and feeds outdoors as well as indoors, vector control methods targeting the human home, such as indoor residual spraying (IRS) and insecticide treated nets (ITNs, or long-lasting insecticidal nets, LLINs), are not sufficient to control this vector, necessitating the use of additional approaches.

With high aridity and low population density, much of the environment of the Arabian Peninsula is unsuitable for the malaria parasite. However, perennial mountain streams support numerous fertile valleys and oases in the southern provinces of Saudi Arabia, where human communities and vector populations coincide, and from where most malaria cases are reported.

Throughout the 1980s, there were a relatively consistent number of confirmed malaria cases in the country, ranging from approximately 5,000 to 17,000 annually. Cases were largely confirmed by microscopy. In 1990, recognising the importance of imported malaria, Saudi Arabia began to distinguish between autochthonous acquired and imported malaria cases by classifying patients according to their travel history, which revealed that imported malaria constituted the vast majority of annual cases.

**Figure 1:**
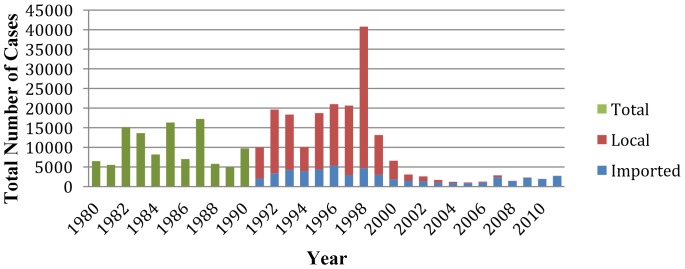
Number of malaria cases in Saudi Arabia 1980 to 2011.

Prior to 1998, national incidence ranged from 0.33/1,000 to 0.96/1,000. In 1998, a major outbreak of autochthonous acquired malaria occurred, in which the total number of confirmed cases in the country reached 36,139. This doubled the total annual incidence to 1.87/1,000 compared to0.92/1,000 in the previous year (Figure 1). Certain areas of the country were disproportionately affected by the outbreak, and incidence in the regions of Jazan and Qunfudah increased from 10/1,000 to 20/1,000 during the outbreak and from 12/1,000 to 44/1,000, respectively [9].

**Figure 2:**
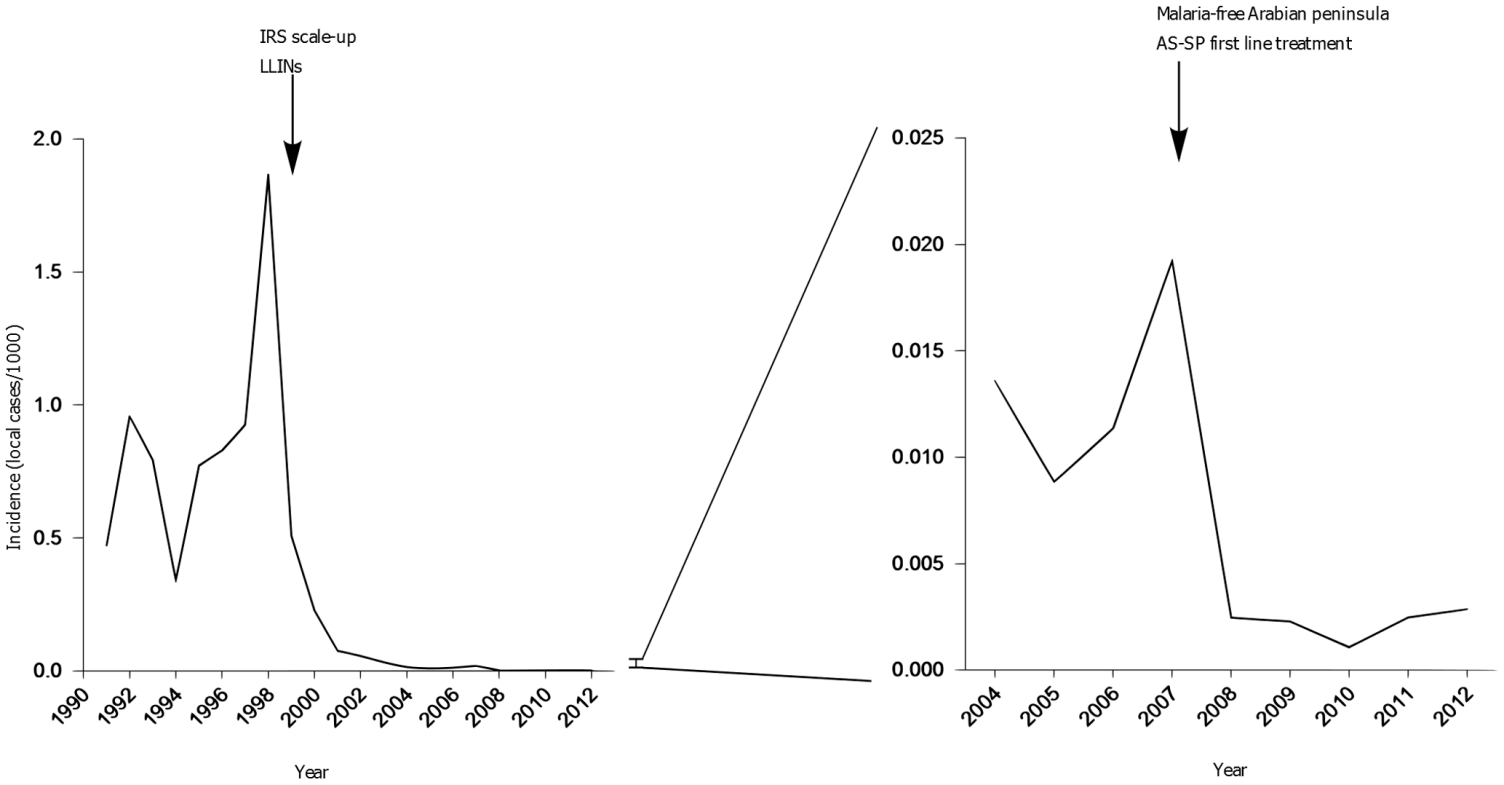
Annual parasite incidence of autochthonous malaria cases with timing of major control milestones indicated. IRS: indoor residual spraying, LLIN: long-lasting insecticidal nets, AS-SP: sulfadoxine pyrimethamine. Although the plan for a malaria-free Arabian peninsula was established in 2001, it was not funded until 2007.

The 1998 outbreak triggered an aggressive centrally co-ordinated control campaign, the result of which was a steep decline in autochthonous transmitted malaria until 2004. During the 2007–2008 season, a further decrease of nearly an order of magnitude occurred from 0.02/1,000 to 0.002/1,000 (Figure 2).

**Figure 3:**
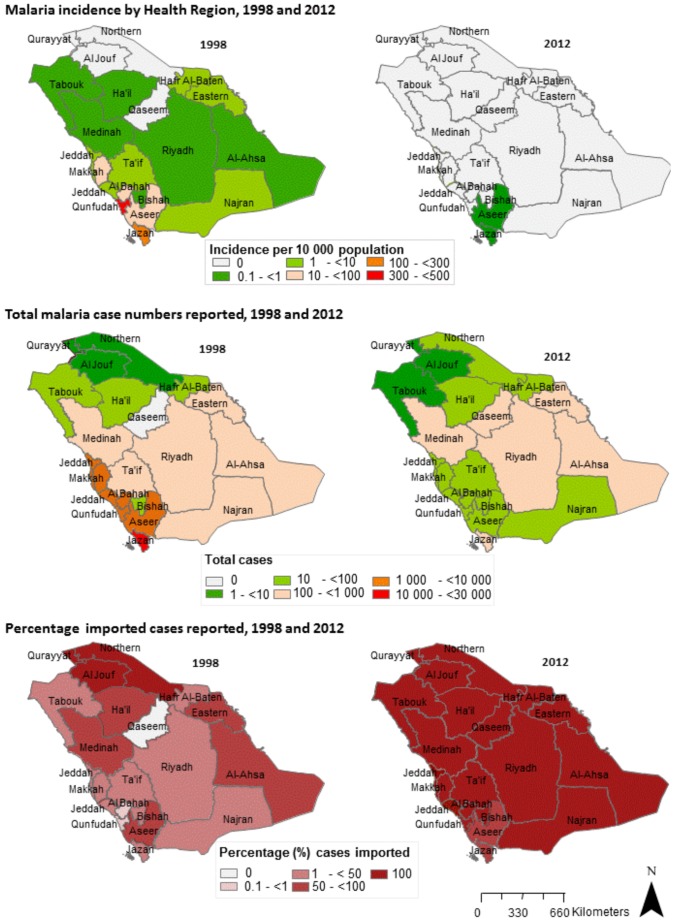
Maps showing the changes in incidence of autochthonous cases, the total number of cases and percentage of imported cases.

**Figure 4:**
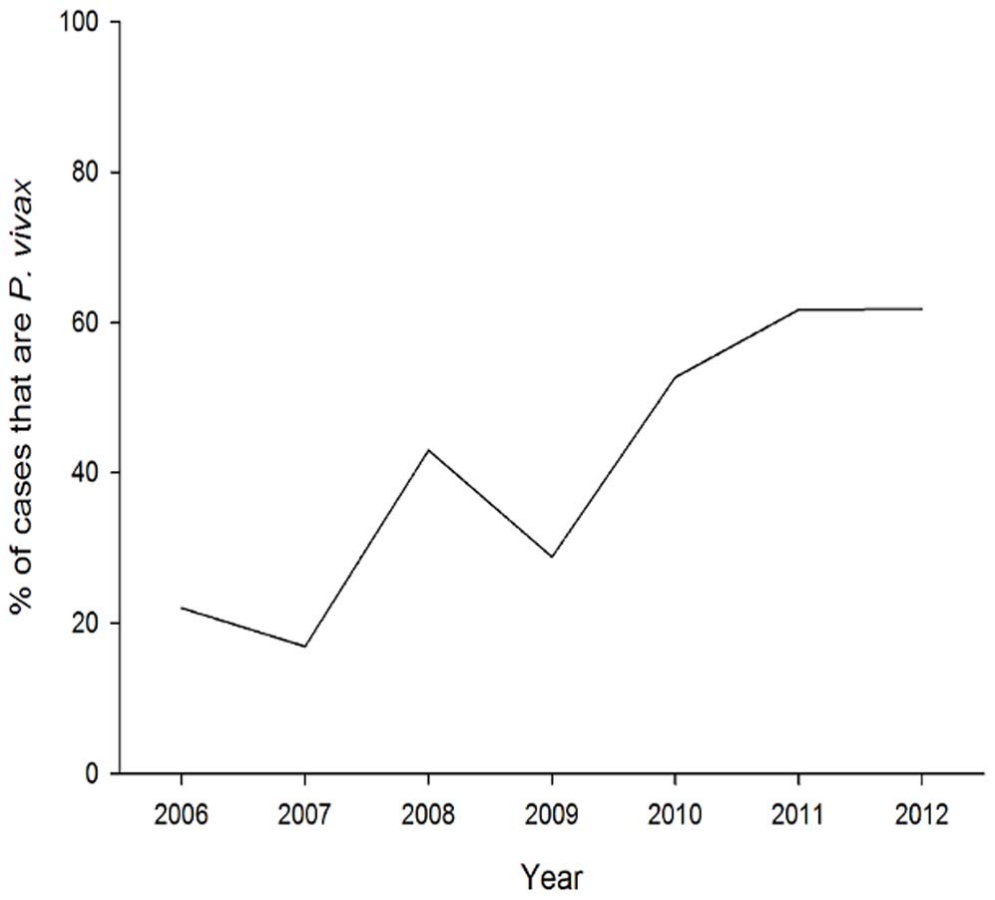
Proportion of confirmed cases caused by *Plasmodium vivax* from 2006–2012.

As the incidence of autochthonous cases has fallen, the proportion of total imported malaria cases has steadily increased from 11% in 1998 to 98% in 2011. By 2010, only two health regions, Aseer (incidence of 0.001/1,000) and Jazan (incidence of 0.013/1,000), recorded any autochthonous malaria cases, and only two malaria-attributable deaths resulting from autochthonous transmission were recorded nationally in 2011. The latest region in Saudi Arabia to eliminate malaria was Qunfudah, which has reported no autochthonous transmission since 2009 (Figure 3) [13]. Additionally, a shift in the predominant parasite species has occurred in the last seven years, from predominantly *P. falciparum* to a majority of the imported *P.vivax* species (Figure 4) [9].

### Control and elimination activities

The current elimination strategy in Saudi Arabia focuses mainly on: a) targeting high risk areas for sustained preventative measures such as long lasting insecticide treated nets (LLINs) and indoor residual spraying (IRS); b) management of infection through rapid confirmed diagnosis and treatment; c) individual case follow up and reactive surveillance with appropriate treatment and vector control and, d) active case detection at borders with screening and treatment. The details of this strategy are discussed below.

#### A. Sustained vector control targeting high-risk areas

Due to the low incidence of malaria, comprehensive vector control is not considered to be cost effective, and is instead guided by case detection for targeted control measures [14], [15]. This maintains coverage of interventions to interrupt transmission in the populations at risk [16], [17]. A total of 68 active transmission foci were recorded in Saudi Arabia in 2011 [13]. The vector control programme in these areas used IRS, distribution of LLINs, larviciding and space spraying in an integrated manner.

DDT-based IRS was introduced in the Eastern Province in 1948 by ARAMCO to protect its workforce. DDT use in the ARAMCO and national programmes continued until 1954, when resistance was detected in *An. stephensi*. This prompted a switch in insecticide to dieldrin for three years before dieldrin resistance was detected in 1957 [18]. During this period, malaria cases decreased from 2000 in 1947 to 54 in 1956 [5] and the ARAMCO IRS campaign became the model for a national programme extended to all malarious areas of the country. In 1963, the government and WHO jointly started a pre-eradication campaign, and by the mid 1970's, IRS with DDT or dieldrin was used as the primary strategy to eliminate foci of transmission throughout the eastern and central regions of the country. Despite this, active transmission persisted in southern regions of the country and along the Red Sea coast. Following a decline in IRS efforts in the late 1970's, targeted IRS with DDT was re-introduced in Jazan and other southern regions in the early1980's [19]. IRS was then scaled back in 1987 when the organophosphate, fenitrothion, replaced DDT as the number of autochthonous cases declined.

Following the 1998 epidemic, IRS activities were scaled up again in southern Saudi Arabia. Focused IRS with DDT was carried out in 1999 at the beginning of peak malaria transmission (April to October). However, it became apparent that DDT resistance still existed, necessitating a switch to the pyrethroid lambda-cyhalothrin (10% EC) in 1999 and deltamethrin (25%WB) in 2004. By 2011, IRS with either deltamethrin (Kaothrine 25 WG) or lambda-cyhalothrin (ICON or Demand 10CS) protected 83% of the 3.15 million persons at risk [20] (Table 1).

**Table 1:** Coverage of ITNs and the number of persons protected by IRS.

Year	Number ITNs distributed	Persons protected by IRS
2004	460,000	unknown
2005	81,364	unknown
2006	0	94,350
2007	0	unknown
2008	250,000	unknown
2009	250,000	2,457,965
2010	81,050	2,500,000
2011	100,000	2,600,000

Source: World malaria reports [20], [48].

Treatment of vector breeding sites with insecticide was introduced as a component of integrated vector control in 1971, initially with Paris Green and then with temephos (EC50). Combined with source reduction, weekly larviciding of 10–15 km sections of the affected wadis (seasonal river valleys) with temephos was performed [6]. Following detection of resistance in 2000, temephos was replaced by larvicides using insect growth regulators, predominantly difubenzuron, methoperene and pyriproxyfen. Larviciding remains one of the primary vector control strategies used in the active transmission foci in Saudi Arabia. Space spraying was used in the early control campaigns to rapidly reduce adult mosquito vector densities around wadis [6]. As this was the only breeding site, this was a successful strategy. Today, space spraying using the pyrethroid insecticides bifenthrin, bioallethrin and cyphenothrin remains a key component of vector control [21]. Currently it is used in response to the detection of a malaria case, when spraying in and around the index home and six nearest neighbours is performed. Following a successful trial of permethrin insecticide treated nets [22], LLINs were added to the national control programme in 1999, with free distribution to all age groups in focal areas. Since 2008, over 680,000 alpha-cypermethrin LLINs (Interceptor, BASF, Germany) have been distributed during the scale up for elimination (Table 1).

**Figure 5:**
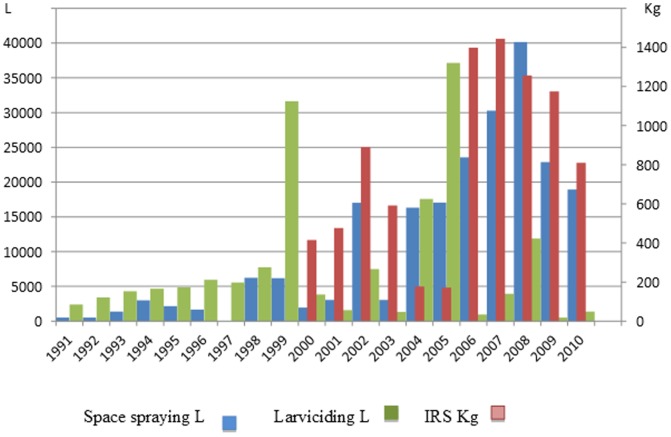
Amount of insecticide used for vector control from 1991 to 2011.

Post 1998, the efforts and resources dedicated to malaria control and elimination are shown in the sustained quantities of insecticide used (Figure 5). The current control programme delivers a range of insecticides from different classes, although pyrethroids are predominantly used against the adult insects. Although the importance of insecticide resistance as a major barrier to the success in malaria control and elimination is well known, [23] no routine insecticide susceptibility was undertaken on the malaria vectors in Saudi Arabia.

#### B. Rapid confirmed diagnosis and treatment

Diagnosis and treatment of malaria has been provided free in Saudi Arabia since the inception of the malaria programme [24], [25]. All cases are confirmed positive by microscopic examination [26]. However, microscopy facilities are not always available (especially with active case detection). In these situations, a rapid diagnostic test (RDT) is used to confirm cases. A blood smear is always taken and read when conditions permit as a measure of quality assurance.

With the detection of chloroquine resistance in *P. falciparum* [27], artesunate plus sulfadoxine-pyrimethamine was made the first line treatment in 2007, with artemether-lumefanthrine being used for complicated malaria and quinine used for severe complicated malaria. Chloroquine plus primaquine are used for *P. vivax*. As ACTs kill developing gametocytes and reduce malaria transmission [28], [29], this treatment strategy is of benefit in areas of low transmission, such as Saudi Arabia, where mature gametocytes may account for residual transmission and outbreaks [30].

#### C. Reactive case detection

Malaria is a notifiable disease in Saudi Arabia and diagnosis is predominantly based on microscopy [25]. Most malaria is identified through passive case detection at health facilities. Since 1991, all cases are reported within 24 hours to the local malaria centre, using a standard form that triggers a follow-up analysis to determine the probable source of infection. Based on the concept that malaria cases tend to cluster [31]–[33], reactive case detection focuses on other members of the household and neighbouring houses. Occupants are tested and treated for malaria, and appropriate space spraying and/or larviciding vector control is performed. This rapid action minimises the potential for any onward transmission from an index case. A similar system has been applied successfully in South Africa [34].

#### D. Active case detection at the border

As autochthonous endemicity is reduced or eliminated, controlling imported malaria becomes more important [4], [35]. While there has been a marked reduction in the actual number of imported malaria cases in recent years (4,657 and 2,719 cases in 1998 and 2011, respectively), the proportion of imported cases has increased dramatically (11.4% and 97.5% of malaria cases in 1998 and 2011 were imported, respectively) [9]. Therefore, there is a clear risk of human movement reintroducing malaria into areas of elimination and creating outbreaks in Saudi Arabia as seen elsewhere in other countries [36]–[38].

There are three main sources of imported malaria in Saudi Arabia. First, Saudi Arabia relies on a large expatriate work force, many of which originate from malaria endemic countries in the Middle East, Africa or Asia. Second, Saudi Arabia contains the most important Islamic holy sites, visited by many millions of pilgrims from every country worldwide. The public health importance of imported malaria and other diseases from pilgrims attending the Hajj has long been recognised [39], [40], and reactive case detection is used as the primary method of limiting transmission in these groups. Lastly, and perhaps most importantly, Saudi Arabia shares a southern border with a malaria endemic country, the Republic of Yemen [20].

Yemen has the second highest incidence of malaria in the Mediterranean region, with an estimated 40% of paediatric admissions due to malaria in some settings [20], [41]. The highest incidences of malaria in Yemen occur in the Tihama coastal plain, a hot and humid geographical region where *An. arabiensis* is common [11], [42], which extends north into Saudi Arabia's Jazan province. High levels of human movement occur between both countries with an estimated 3,000 illegal immigrants crossing from Yemen to Saudi Arabia on a daily basis and some 20,000 Saudi Arabians spending weekends in Yemen [43], [44].

To limit the risk of imported malaria, plans were established in 2001 for a malaria-free Arabian peninsula by 2020 [38]. The plan was endorsed in 2005 by the Eastern Mediterranean Regional Office of WHO [45] and by the Health Ministers of the Gulf Cooperation Council in 2007, with financial support of $42.7 million [41]. Elimination in the Arabian Peninsula has been largely successful, and only Saudi Arabia and Yemen have yet to achieve malaria free status [46].

Cross border collaboration between Saudi Arabia and Yemen has included the establishment of malaria centres offering free screening and treatment, mostly for Yemenis living in border villages. These are supported by mobile teams that carry out active case detection to target high risk populations living in the border villages [47]. These mobile teams detected ∼50% of imported cases in 2010–11 in Jazan. Malaria control in Yemen includes joint Saudi-Yemeni vector control teams, which are responsible for IRS and for space-spraying a 10 km wide buffer zone inside Yemen. Unfortunately, recent political unrest and a deteriorating political situation in Yemen have halted these efforts.

## Discussion

Since the outbreak in 1998, the efforts to control and eliminate malaria in the Kingdom of Saudi Arabia have produced significant gains. The total number of cases has remained consistently low since 2001 and the number of autochthonous cases has decreased from 467 in 2007 [48] to just 82 in 2012 [13]. With incidence already well below 0.01 cases per 1,000 persons, elimination of malaria in Saudi Arabia is a realistic goal. However, a number of considerable challenges must be overcome in order to achieve this target.

Migration of humans (and parasites) has been shown to rapidly undermine gains made in malaria control efforts [49], [50], and in Saudi Arabia, imported malaria still remains a major problem. The border with Yemen is an area of central concern, and controlling malaria in this region is a major challenge for Saudi Arabia in achieving and maintaining a malaria free status [46]. Autochthonous transmission is limited due to an effective response by the malaria control programme and/or unfavourable conditions for an outbreak to occur. However, the number of autochthonous cases increased to from 29 in 2010 to 69 and 82 in 2011 and 2012, respectively. This, in part, is probably due to the disruption of malaria control activities on the Yemeni side of the border and an increase in both legal and illegal immigrants into Saudi Arabia. Continued importation combined with the presence of vectors means that autochthonous transmission will inevitably occur. Saudi Arabia must find ways to mitigate this risk and ensure that endemic transmission is halted.

Saudi Arabia is likely to experience further difficulties as the proportion of cases attributable to *P. vivax* increases. *P. vivax* is generally less responsive to control than *P. falciparum* for a number of reasons. First, dormant liver stages can lead to relapse. Second, the extrinsic incubation period is shorter. Third, infective gametocytes are produced concurrently with asexual stages. Fourth, parasite densities are often lower than the detection threshold of diagnostic tests. Lastly, adherence to the 7–14 day primaquine regimen to treat liver stages is often poor. All of these factors make *P. vivax* residual transmission more difficult to detect and control.

### The way forward

Regional efforts such as the Lubombo Spatial Development Initiative [35] and the Asia Pacific Malaria Elimination Network [51], [52] are excellent examples of where cross border collaborations have succeeded in addressing the challenge of imported malaria. Today, seven countries (Bahrain, Kuwait, Oman, Qatar, Saudi Arabia, the United Arab Emirates and Yemen) remain committed to supporting intensification of malaria control efforts on the Arabian Peninsula [20]. It is clear that reinvigoration of the border collaboration with Yemen will be necessary to achieve a malaria-free Saudi Arabia by 2015. Novel strategies of quickly identifying imported cases should also be considered. Recent work in Swaziland, a country also targeting elimination by 2015, has shown that snowball and time-location sampling can quickly identify networks of individuals at high-risk of bringing malaria into the country [53]. Adoption of this approach in Saudi Arabia would allow for targeted screening and treatment, preventing onward transmission. Additional strategies may include parasite genotyping, which would allow more accurate determination of common sources of infection.

Preventing the growing proportion of P.vivax cases will require implementing new but currently available strategies to quickly identify and treat those infected with the parasite. This may include sensitive, point-of-care diagnostics that are compatible with field conditions, such as a loop-attenuated isothermal amplification assay (LAMP). This assay is as sensitive as PCR detection, and suitable for deployment in remote settings has been shown to be equally as sensitive as PCR, but able to be deployed in remote settings [54].

Preventing autochthonous transmission requires a robust and sophisticated information system that enables timely response to outbreaks. Saudi Arabia aims to strengthen their control programme using emerging information technologies that can inform elimination efforts and maintain the disease free status. It is currently enhancing its surveillance systems through the adoption of a Disease Data Management System, a spatial decision support tool [55] that has a focus on malaria clusters and outbreaks [34], [56] with mapping functionality required to support elimination and ongoing surveillance by mapping down to the village and even household level as recommended by WHO [57]. This will enhance the control programme's ability to investigate disease clusters and adjust operational plans accordingly. An independent review recently highlighted the advantages of such a system [58]. The failure to maintain high quality surveillance after near-elimination was a key factor that resulted in the disease returning in India [59] and Madagascar highlands [60].

Malaria elimination is a surprisingly stable state [61]. However, sustained elimination is unlikely to be attributable to continuous vector control. Rather, economic development that reduces vector-human contact and improved health systems are the major drivers in transitioning from malaria-endemic to malaria-free. As such, while improved management of imported malaria, more rapid case detection, and highly targeted interventions should be encouraged in the short-term, investment in health system infrastructure and overall socioeconomic development should be envisioned as the driving force behind malaria elimination in-country and regionally. Given the continued political and financial support from the government, the prospects to attain and sustain elimination in Saudi Arabia are positive.
